# Special Section Guest Editorial: Tissue Phantoms to Advance Biomedical Optical Systems

**DOI:** 10.1117/1.JBO.27.7.074701

**Published:** 2022-06-27

**Authors:** Dimitris Gorpas, Heidrun Wabnitz, T. Joshua Pfefer

**Affiliations:** a Institute of Biological and Medical Imaging, Helmholtz Zentrum München, Neuherberg, Germany; b Technical University of Munich, Chair of Biological Imaging, Munich, Germany; c Physikalisch-Technische Bundesanstalt (PTB), Berlin, Germany; d Food and Drug Administration, Silver Spring, Maryland, United States

## Abstract

The editorial introduces the JBO Special Section on Tissue Phantoms to Advance Biomedical Optical Systems.

Over the past 40 years, tissue-simulating phantoms have exhibited their utility in a broad range of roles for biomedical optics technologies.^1^^,^^2^ Early in the device life cycle, these roles include new method/application proof-of-principle, elucidation of light–tissue interaction phenomena, design optimization, establishment of device viability prior to performing *in vivo* studies, and clinical trial standardization. Later in the product life cycle, phantoms can enable device inter-comparisons, detect deviations in performance, facilitate evaluation of post-market adverse events, accelerate clinician training, and streamline calibration and repair.

Simple homogeneous phantoms were initially created as a way to test theories and models to establish the feasibility of new techniques using controlled experiments in materials possessing known optical properties and structure.^3^ Today, many phantoms incorporate biologically-relevant features such as dynamic functionality, specialized optical properties (e.g., birefringence), or anthropomorphic geometry. Yet a large base of use still centers on diffuse optical spectroscopy and imaging approaches in which the primary phantom requirements are optical property and geometric accuracy, repeatability, lifetime, and reliability for standardization and consistent implementation under realistic conditions.

When well-validated for a specific purpose, phantom-based test methods can be incorporated into international consensus standards used to guide device development and regulation. These standards can facilitate clinical translation and commercial success, as well as provide an indicator of a technology’s maturity. For example, an international standard for pulse oximeters has been in existence for many years,^4^ while more recent efforts have culminated in standards for cerebral oximetry^5^ and functional near-infrared spectroscopy.^6^ Furthermore, standardization activities for other areas, such as fluorescence imaging^7^^,^^8^ and photoacoustic imaging^9^^,^^10^ are also in progress. Advances in tissue phantoms benefit these efforts towards international standards in Biomedical Optics that will further boost the clinical translation of different technologies within this field.

This JBO Special Section encompasses a diverse range of studies in basic research, development, and application of tissue-simulating phantoms. A wide variety of modalities are represented as well, from optical coherence tomography and Raman spectroscopy to diffuse optical spectroscopy, photoacoustic imaging, and fluorescence imaging. We begin our introduction of this section with two synthesis articles. One of these focuses on photoacoustics—while also covering phantoms in established medical imaging modalities outside of optics—and the other, on fluorescence imaging.

With the impressive developments in optical phantoms in recent years, it is easy to overlook the fact that the inspiration for these approaches often derives from more well-established medical imaging modalities such as ultrasound, X-ray, CT and MRI. Image quality test methods for these modalities are described in international consensus standards, including phantom designs, materials (matrix and inclusion), and testing procedures/calculations. A recent article by Palma-Chavez et al.^11^—initially submitted to this Special Section but published early (Vol. 26)—provides an extensive review of these documents. This article discusses how some techniques have already been adapted for photoacoustic imaging, and provides recommendations for future efforts. By leveraging successful approaches, the field can more rapidly achieve effective tools for clinical translation of optical systems.

Fluorescence endoscopy is another powerful modality, as it can provide molecular targeting for hard-to-detect gastrointestinal cancers, ideally through quantitative imaging. In their Perspective article, Sterkenburg et al. [https://doi.org/10.1117/1.JBO.27.7.074704] review scientific literature relevant to standardization of this technique, including fluorescence phantoms for performance testing. The authors describe phantoms that can provide thorough image quality characterization or streamlined day-to-day testing, then conclude with a discussion of exciting new directions for this technology.

The exploration of various phantom materials and designs as well as phantom characterization is the central topic of seven of the research articles in this Special Section.

Standardized testing can only be effective if the phantoms used are consistent and their properties accurately benchmarked. Zhao et al. [https://doi.org/10.1117/1.JBO.27.7.074713] analyzed two matrix materials for diffuse optical spectroscopy and imaging commonly used to achieve a high degree of stability—epoxy resin and silicone. The authors performed optical property measurements with three time-resolved diffuse spectroscopy systems, which provided insights into reproducibility of these phantom materials as well as limitations of techniques used for reference measurements.

Fabrication of silicone phantoms was also addressed by Goldfain et al. [https://doi.org/10.1117/1.JBO.27.7.074706] who report on a robust technique to fabricate silicone-based phantoms with widely and independently adjustable scattering and absorption properties. The spectra of absorption and scattering coefficients were measured with a broadband integrating sphere system and characterized correspondingly with two-parameter linear and exponential spectral fits. These manufacturing and analysis techniques may further promote the application of silicone tissue phantoms and their incorporation into emerging standards.

Modifications of well-established tissue-mimicking materials can sometimes yield significant new applications. Chang et al. [https://doi.org/10.1117/1.JBO.27.7.074711] studied the birefringence of silicone and found variations as a function of tensile deformation. This relationship was leveraged to achieve phantoms that provided biologically relevant signals for polarization sensitive optical coherence tomography (PS-OCT), with a focus on bladder cancer. Such an approach can aid clinical training and standardized evaluation of polarization-based devices.

Hydrogel phantoms are commonly used to represent biological tissue when accurate simulation of the spectral characteristics of blood is required. In the study by Jonasson et al. [https://doi.org/10.1117/1.JBO.27.7.074712], hydrogel phantoms were fabricated to simulate inflammation and edema by incorporating a range of water fractions. Broadband sample characterization was performed by a visible-NIR spatial frequency domain imaging system. This article also discusses the advantages and disadvantages of different types of gelatin and chromophores, so as to assist researchers in identifying the optimal approach for specific tasks.

In another study of phantoms that provide realistic wide-band spectral properties of blood, Majedy et al. [https://doi.org/10.1117/1.JBO.27.7.074708] compared the spectral signatures of three chromophores (whole blood and two types of hemoglobin solutions). Measurements across the visible range in liquid phantoms showed faithful recapitulation of oxygenated blood absorption. However, representation of the de-oxygenated blood spectra was less accurate, due in part to methemoglobin. Additionally, the authors noted that oxygen diffusion at the air interface can impact superficial saturation levels.

Innovative test methods can accelerate progress of new optical modalities and applications, particularly when physiological properties are critical to device performance. Pacheco et al. [https://doi.org/10.1117/1.JBO.27.7.074707] present a unique phantom with dynamic capabilities for near-infrared spectroscopic gas sensing in the neonatal lung. The rigorous model developed in this study has the potential to elucidate the capability of a technology currently under clinical study and establish feasibility under realistic conditions.

Three-dimensional printing is a powerful technology that can serve as a platform for the fabrication of innovative development tools such as anatomically realistic phantoms. Research by Schädel-Ebner et al. [https://doi.org/10.1117/1.JBO.27.7.074702] has resulted in a tissue-simulating material for 3D printing with indocyanine-green-simulating spectral features that are highly stable. After rigorous characterization, this material was used to print anthropomorphic phantoms for rheumatoid disease imaging in the hand.

Another important aspect of standardization in Biomedical Optics is, of course, the application of phantoms at different stages of the medical device development process. The following seven articles are focused on optimizing system performance characterization for diffuse optics and other modalities.

Multi-laboratory initiatives are essential to establish protocols and develop reference tissue phantoms that are universally effective. Lanka et al. [https://doi.org/10.1117/1.JBO.27.7.074716] present the largest multi-laboratory performance assessment and comparison of diffuse optics instrumentation, involving 28 instruments in 12 institutions. Eight tests based on three consolidated protocols (BIP, MEDPHOT, nEUROPt)^12^^–^^14^ were implemented on three kits of tissue phantoms. The overall assessment of the set of results was based on 20 synthetic indicators, in part newly defined. Such tests can be employed to assess the impact of hardware implementations or to compare different analysis tools.

Ban et al. [https://doi.org/10.1117/1.JBO.27.7.074710] employed a subset of tests of the same three protocols (i.e., BIP, MEDPHOT, and nEUROPt) to characterize the performance of a the newly developed advanced, miniaturized, modular and wearable multi-channel time-domain functional near-infrared spectroscopy (TD-fNIRS) system for localizing and quantifying brain activation in humans. The system was shown to compare well with existing research-grade TD-fNIRS systems. This is an exemplary study of how phantom-based standardized protocols can be employed to validate and characterize technological advancements in Biomedical Optics that lead to product development, clinical translation, and opening up new application areas.

Performance constancy for optical devices is critical for clinical studies involving multiple sites. Towards this goal, Schmidt et al. [https://doi.org/10.1117/1.JBO.27.7.074714] evaluated the use of phantoms for ensuring consistent reflectance measurements with fiberoptic probes over time. Specifically, this was accomplished by measuring the optical properties of the samples using diffuse reflectance and comparing key metrics derived from these results. Both laboratory and clinical measurements generated in this work provide evidence of the potential utility of this approach for multi-center trials.

Raman spectroscopy has numerous biomedical applications and is on the verge of clinical translation, yet a lack of standards for *in vivo* systems represents an obstacle to achieving its full potential. Fales et al. [https://doi.org/10.1117/1.JBO.27.7.074705] characterized three different Raman systems based on four standardized (non-phantom) protocols, revealing great inter-system variations in resolution, sensitivity, and stray light rejection. However, using a custom 3D-printed phantom with bio-relevant Raman properties, the authors showed that all three systems surprisingly achieved similar performance as a function of depth. This finding is significant in that it demonstrates that Raman system specifications are not sufficient to assess performance, rather phantoms should also be used to provide more practical estimates.

Phantom-based performance assessment is also crucial for quantitative imaging methodologies. To assess relevant image quality characteristics of 3D hybrid photoacoustic-ultrasound breast imaging systems, Dantuma et al. [https://doi.org/10.1117/1.JBO.27.7.074709] developed five dedicated 3D test objects. Their designs and production protocols were presented, together with applications to study various performance measures, e.g., spatial resolution for photoacoustics and ultrasound. With such a standardized approach, system settings and measurement protocols can be optimized prior to human studies.

In fluorescence-guided imaging for surgical intervention there is a need for well-validated reference materials and corresponding methods to quantify fluorescence radiation. Litorja [https://doi.org/10.1117/1.JBO.27.7.074715] developed a method to calibrate a fluorescence imager, i.e., convert imager-specific arbitrary digital counts to the fluorescence radiant flux detected. This method relies on a fluorescing tissue phantom that is calibrated to be traceable to the international system of units. The article describes the radiometric method and presents an example of an imager calibration.

While conceptualization of phantoms often originates with engineers, clinicians can be an important resource for innovation as well. Neonatologist and Professor of Pediatrics Gorm Greisen has contributed a unique Perspective article [https://doi.org/10.1117/1.JBO.27.7.074703] with a clinically focused challenge for phantom developers. Dr. Greisen discusses the need for digital and physical phantoms to explore a novel method of cerebral oximetry in newborns. The measurement of venous oxygenation in the superior sagittal sinus through the fontanelle promises advantages over established cerebral tissue oximetry. According to this perspective, the steps to identify an optimal optode arrangement, evaluate sensitivity, and optimize algorithms should also include the design of phantoms to represent the anatomy and its variation.

Finally, as Guest Editors, we are grateful for the impressive contributions to this Special Section by experts in diverse areas of Biomedical Optics. Our field has witnessed an immense advance during the last few years, with many technologies undergoing clinical validation studies or entering the regulatory approval process. This progress has brought standardization into the spotlight of research and been accompanied by great strides in structural and functional bio-realism of phantoms. Articles presented in this Special Section represent significant contributions to this effort, and we are enthusiastic about the potential for the achievements described here to yield significant impacts on the field for many years to come.
